# Serum 25-hydroxyvitamin D and cognitive decline in the very old: the Newcastle 85+ Study

**DOI:** 10.1111/ene.12539

**Published:** 2014-08-13

**Authors:** A Granic, T R Hill, T B L Kirkwood, K Davies, J Collerton, C Martin-Ruiz, T von Zglinicki, B K Saxby, K A Wesnes, D Collerton, J C Mathers, C Jagger

**Affiliations:** a Institute for Ageing and Health, Newcastle UniversityNewcastle upon Tyne, UK; b Human Nutrition Research Centre, Newcastle UniversityNewcastle upon Tyne, UK; c School of Agriculture, Food and Rural Development, Newcastle UniversityNewcastle upon Tyne, UK; d Wesnes Cognition LtdStreatley-on-Thames, UK; e Department of Psychology, Northumbria UniversityNewcastle upon Tyne, UK; f Institute of Neuroscience, Newcastle UniversityNewcastle upon Tyne, UK

**Keywords:** attention, cognitive decline, cohort study, 25-hydroxyvitamin D

## Abstract

**Background and purpose:**

Studies investigating the association between 25-hydroxyvitamin D [25(OH)D] and cognition in the very old (85+) are lacking.

**Methods:**

Cross-sectional (baseline) and prospective data (up to 3 years follow-up) from 775 participants in the Newcastle 85+ Study were analysed for global (measured by the Standardized Mini-Mental State Examination) and attention-specific (measured by the attention battery of the Cognitive Drug Research test) cognitive performance in relation to season-specific 25(OH)D quartiles.

**Results:**

Those in the lowest and highest season-specific 25(OH)D quartiles had an increased risk of impaired prevalent (1.66, 95% confidence interval 1.06–2.60, *P *=* *0.03; 1.62, 95% confidence interval 1.02–2.59, *P *=* *0.04, respectively) but not incident global cognitive functioning or decline in functioning compared with those in the middle quartiles adjusted for sociodemographic, health and lifestyle confounders. Random effects models showed that participants belonging to the lowest and highest 25(OH)D quartiles, compared with those in the middle quartiles, had overall slower (log-transformed) attention reaction times for Choice Reaction Time (lowest, *β *= 0.023, *P *=* *0.01; highest, *β *= 0.021, *P *=* *0.02), Digit Vigilance Task (lowest, *β *= 0.009, *P *=* *0.05; highest, *β *= 0.01, *P *=* *0.02) and Power of Attention (lowest, *β *= 0.017, *P *=* *0.02; highest, *β *= 0.022, *P *=* *0.002) and greater Reaction Time Variability (lowest, *β *= 0.021, *P *=* *0.02; highest, *β *= 0.02, *P *=* *0.03). The increased risk of worse global cognition and attention amongst those in the highest quartile was not observed in non-users of vitamin D supplements/medication.

**Conclusion:**

Low and high season-specific 25(OH)D quartiles were associated with prevalent cognitive impairment and poorer overall performance in attention-specific tasks over 3 years in the very old, but not with global cognitive decline or incident impairment.

## Introduction

Recent evidence from life sciences and epidemiology points to the role of serum 25-hydroxyvitamin D [25(OH)D] in brain function, including cognition, across the life span 1,2. Detection of hydroxylases for vitamin D activation and vitamin D receptors in neurons and glia in brain regions essential for cognition and memory implicates their relevance for brain health. Moreover, *in vitro* and *in vivo* studies propose neuroprotective properties of 25(OH)D 2.

Current epidemiological research suggests an inverse or non-linear (i.e. curvilinear or U-shaped) association between circulating 25(OH)D concentration and risk of several age-related chronic diseases and all-cause mortality 3–8, suggesting beneficial health outcomes at moderate (∽50–60 nM) or higher concentrations (∽75–80 nM). Based on skeletal health 7, it has been estimated that 50 nM (20 ng/ml) of 25(OH)D meets the requirements of >97.5% of the US population across all age groups. However, a number of studies have demonstrated a high prevalence of 25(OH)D inadequacy amongst older adults based on a clinical threshold of 25 or 30 nM 9,10. A higher risk of low vitamin D status (<25 nM) in older adults has been linked to reduced epidermal stores of 7-dehydrocholesterol (vitamin D precursor), immobility, malnutrition, renal impairment and environmental factors 10,11. However, current scientific evidence is inadequate to allow the development of recommendations for optimal age-specific 25(OH)D concentrations, or clinical thresholds, for extra-skeletal health in older adulthood. There is also uncertainty about the utility of vitamin D supplementation, especially for those aged 75+ living with comorbidities 7,11,12. Although recent evidence indicates that supplementation improves vitamin D status in older adults without adversely affecting heath and survival 13, there is no consensus on the definition of hypovitaminosis D and upper 25(OH)D thresholds for optimum physical and mental health in old age to prevent problems with under- or over-treatment 7,11,12,14.

Non-optimal concentration of 25(OH)D, variously defined as <25 nM (10 ng/ml) or <50 nM (20 ng/ml), has been implicated as a risk factor for global cognitive impairment 15,16 and weaker performance on domain-specific cognitive tasks 17–20 in several, but not all, cross-sectional studies 21 involving adults aged 60+. Only four prospective studies reported an increased risk of cognitive decline in association with lower concentrations of serum 25(OH)D (≤50 nM) in adults aged 65+ 22–25. Studies on 25(OH)D and cognitive decline in those aged 85+ are scarce 26,27.

The Newcastle 85+ Study was therefore used to test for the presence of either an inverse or a non-linear association between 25(OH)D concentrations and cognition at baseline and cognitive decline over 3 years, utilizing measures of global and attention-specific cognitive function.

## Method

### Participants

Participants were drawn from the Newcastle 85+ Study, a longitudinal study of health trajectories and outcomes in a single-year birth cohort (1921) recruited from general practices in Newcastle and North Tyneside, UK, as described elsewhere 28,29. The study was approved by the Newcastle and North Tyneside Local Research Committee One. At baseline (2006−2007), both multidimensional health assessment and general practice records data were available for 845 individuals who formed the basis for this analysis. Serum 25(OH)D was successfully measured from blood samples in 775 (91.7%) individuals 30. Participants were followed up at 1.5 and 3 years.

### Cognitive assessments

Global cognitive function was evaluated using the Standardized Mini-Mental State Examination (SMMSE), a brief dementia-screening instrument which provides a global score of cognitive function ranging from 0 to 30 points 31 that correlates well with performance in activities of daily living 31,32. In all, 773 (91.5%) participants with a baseline SMMSE score and 25(OH)D status formed our analytical sample (Fig. S1). Cognitive status was defined as normal (SMMSE scores ≥26) or impaired (SMMSE scores ≤25) 31,32 at baseline and 3-year follow-up. Incident cognitive impairment was defined as crossing the 25-point threshold of the SMMSE 32. Models in which cognitive decline was defined as a loss of ≥3 points from the baseline score (i.e. clinically meaningful or reliable change) 33,34 were also considered.

Attention was measured using the attention subsets of the Cognitive Drug Research (CDR) computerized assessment system 35,36 (see Method S1 for details), comprising mean reaction times (speed scores) of correct responses (in ms) for Simple Reaction Time (SRT), Choice Reaction Time (CRT) and Digit Vigilance Task (DVT). SRT measures alertness and concentration; CRT examines similar abilities including the extra time taken to discriminate between two opposing stimuli (i.e. reflecting the additional information processing in this task); and DVT tests sustained attention whilst ignoring distractors. Additionally three validated composite measures derived from these tasks were used: Power of Attention (PoA), a sum of the three attention speed scores, measures the intensity of concentration and the ability to focus attention 37; Reaction Time Variability (RTV), a sum of coefficients of variance of the three speed scores, measures fluctuation in attention 38,39; and Continuity of Attention (CoA), a combination of the accuracy scores from CRT and DVT, assesses the ability to sustain attention over the testing period 39. Details of how composite measures were constructed and their validation process are described in Method S1. For all attention-specific measures except CoA, lower scores indicate better performance. A total of 761 participants had at least one attention task score at baseline. A training session 1 week prior to baseline measurements was undertaken with 91.5% (*n *=* *773) of participants 40.

### Serum 25(OH)D assay

Serum 25(OH)D was obtained from fasting morning blood samples and measured by the DiaSorin Radioimmune Assay kit as described 30 (see Method S2 for details). The mean (SD) time between cognitive testing and blood sampling was 0.28 (0.59) months. Because of seasonal variation in 25(OH)D concentration 41, the season of blood draw [December–February (Winter), March–May (Spring), June–August (Summer), and September–November (Autumn)] was controlled for by creating season-specific quartiles (SQ1–SQ4) 42 with cut-offs of 5–17 nM (Spring) to 8–20 nM (Autumn) for SQ1, 18–26 nM (Spring) to 29–45 nM (Summer) for SQ2, 27–46 nM (Spring) to 46–68 nM (Summer) for SQ3 and ≥47 nM (Spring) to ≥69 nM (Summer) for SQ4 (for details see Method S3). The middle quartiles (SQ2 and SQ3) were combined and used as the referent, thus generating three season-specific 25(OH)D groups: lowest, middle and highest.

### Other measures/confounders

Confounders used in the above reported studies 16,22,23,25 were considered for inclusion in the models (see Method S4 for details). Briefly, sociodemographic factors (sex, education, income), health and morbidity [individual chronic diseases: cardiovascular diseases (includes hypertension, cardiac disease and peripheral vascular disease), cerebrovascular diseases, diabetes, osteoporosis; or total number of chronic diseases, renal impairment, depression, waist−hip ratio, serum vitamin B_12_ and folate), and lifestyle factors (smoking, alcohol and physical activity) were included. Because the intake of supplements and medication containing vitamin D was regarded as an important biological determinant of 25(OH)D status in this population, separate analyses were conducted with the entire cohort and with a ‘restricted cohort’ (i.e. excluding 167 (19.8%) individuals who were taking vitamin D supplements/medication).

### Statistical analysis

Participants (*n *=* *845) were compared across the three season-specific 25(OH)D groups by Kruskal–Wallis tests for ordered and non-normally distributed continuous variables and *χ*^2^ tests for categorical variables. For several confounders, missing values were imputed to the reference value to allow for comparison of nested models. Several logistic regression models were fitted to explore the association between season-specific 25(OH)D groups and prevalent cognitive impairment (odds ratio, 95% confidence interval). Models were unadjusted (model 1), adjusted for sex and education (model 2) and adjusted for all confounders (model 3). The models were then fitted with incident cognitive impairment defined as converting from normal to impaired cognitive status at 3-year follow-up. Cognitive status 3 years post baseline was also examined, and cognitive decline of ≥3 points as an outcome was used to determine a clinically meaningful/reliable change in the SMMSE 33,34.

All attention reaction times were converted into seconds and logarithmically (log_10_) transformed to correct a positive skew, and to aid convergence. PoA and RTV were also log_10_ transformed, whereas CoA was negatively skewed and corrected as NEWX = SQRT(*K *− *X*) in which *K* = maximum score + 1. Using all available data for 845 participants, multilevel linear modelling 43 was conducted to determine the effect of 25(OH)D on initial level and rate of change over 3 years in attention-specific tasks, and a series of linear growth curve models were fitted as follows: (i) with ‘time’ in the study (to examine the linear trend of time) and season-specific 25(OH)D groups [to test whether initial status (intercept) varied by 25(OH)D] (model 1); (ii) with an interaction of season-specific 25(OH)D groups and time [to test for varying rates of change by 25(OH)D] (model 2); and (iii) with further adjustment for confounders associated with cognition and 25(OH)D levels (model 3). The SPSS MIXED procedure (SPSS, IBM Corporation, Armonk, NY, USA), with restricted maximum likelihood method and autoregressive error covariance matrix was used to generate parameter estimates (*β*) for effects.

A number of sensitivity analyses of the 25(OH)D groups in relation to cognitive outcomes were undertaken. The analyses were repeated defining cognitive change as scoring −1 SD and −1.5 SD below the mean and using ≤23 SMMSE points for cognitive impairment (Method S5). The analyses were also repeated excluding participants with a diagnosis of dementia, and whether findings were due to terminal drop was explored.

## Results

### Baseline characteristics of the participants by 25(OH)D groups

Participants in the middle season-specific 25(OH)D group (SQ2 and SQ3 combined) were the least depressed and the most physically active, whilst those in the highest quartile (SQ4) were more likely to be female, to take prescribed vitamin D, to have osteoporosis, and to have the highest levels of serum folate compared with participants in the other groups (Table S1). Compared with the middle group, those belonging to the lowest (SQ1) and/or highest (SQ4) season-specific 25(OH)D groups had more cognitive impairment (≤25 SMMSE score) (*H*_(2)_ = 26.55, *P *<* *0.001) and had worse reaction times on DVT (*H*_(2)_ = 8.91, *P *=* *0.01), PoA (*H*_(2)_ = 8.74, *P *=* *0.01), RTV (*H*_(2)_ = 7.73, *P *=* *0.02) and CoA (*H*_(2)_ = 10.51, *P *=* *0.005) (Table1).

**Table 1:** Global cognitive and attention scores^a^ of participants in the Newcastle 85+ Study by season-specific 25(OH)D groups^b^ at baseline and follow-up

Cognitive domain/scores	All participants	Lowest season-specific 25(OH)D group	Middle season-specific 25(OH)D group	Highest season-specific 25(OH)D group	*P* value^c^
Cognitive status by SMMSE					
Baseline (*n*)	839	191	392	190	
Total SMMSE (mean, SD)	25.9 (5.3)	25.0 (5.9)	27.0 (3.6)	25.4 (5.9)	<0.001
Impaired (≤25 SMMSE score %, *n*)	28.6 (240)	37.7 (72)	19.4 (76)	33.7 (64)	<0.001
Normal (26–30)	71.4 (599)	62.3 (119)	80.6 (316)	66.3 (126)	
Follow-up at 3 years (*n*)	470	101	248	103	
Total SMMSE (mean, SD)	25.4 (5.5)	24.0 (6.4)	26.1 (4.6)	25.4 (5.6)	0.005
Impaired (≤25 SMMSE score %, *n*)	34.0 (160)	47.5 (48)	29.8 (74)	30.1 (31)	0.004
Normal (26–30)	66.0 (310)	52.5 (53)	70.2 (174)	69.9 (72)	
CDR Attention Battery					
baseline (*n*)	761	177	373	176	
SRT (ms, mean, SD)	475.4 (488.0)	509.1 (540.6)	459.5 (548.4)	470.1 (234.7)	
CRT (ms, mean, SD)	650.6 (350.7)	687.6 (360.6)	623.0 (340.6)	658.0 (251.6)	
DVT (ms, mean, SD)	526.0 (69.9)	534.8 (73.0)	518.5 (65.5)	535.5 (76.0)	0.01
PoA (ms, mean, SD)	1618.3 (583.7)	1700.4 (846.1)	1549.4 (337.6)	1663.4 (490.2)	0.01
RTV (mean, SD)^d^	64.3 (19.9)	66.7 (21.5)	62.7 (19.2)	65.3 (20.1)	0.02
CoA (mean, SD)^e^	51.4 (9.6)	50.3 (9.7)	52.4 (8.4)	51.6 (10.3)	0.005
Follow-up at 1.5 years (*n*)	570	127	284	130	
SRT (ms, mean, SD)	491.4 (314.1)	500.4 (315.0)	471.5 (290.3)	517.3 (357.6)	
CRT (ms, mean, SD)	669.0 (311.5)	698.1 (317.8)	642.4 (290.3)	681.3 (268.7)	0.03
DVT (ms, mean, SD)	532.7 (73.8)	541.8 (70.1)	528.2 (70.7)	532.4 (80.5)	
PoA (ms, mean, SD)	1691.7 (622.6)	1738.4 (653.4)	1642.1 (563.6)	1727.3 (624.0)	
RTV (mean, SD)	64.4 (21.7)	66.7 (24.7)	62.1 (18.0)	65.6 (23.4)	0.02
CoA (mean, SD)	51.6 (8.5)	50.3 (9.8)	52.2 (7.4)	52.3 (8.1)	0.005
Follow-up at 3 years (*n*)	416	87	222	92	
SRT (ms, mean, SD)	475.3 (251.4)	509.5 (303.8)	454.6 (181.9)	498.1 (339.1)	
CRT (ms, mean, SD)	677.9 (368.9)	727.6 (445.2)	649.2 (327.1)	709.2 (403.6)	
DVT (ms, mean, SD)	533.8 (73.6)	540.8 (69.9)	531.9 (72.5)	531.7 (80.9)	
PoA (ms, mean, SD)	1687.5 (620.8)	1777.9 (754.7)	1635.6 (510.5)	1741.3 (745.2)	
RTV (mean, SD)	63.4 (21.1)	62.2 (17.9)	62.6 (21.8)	65.2 (21.5)	
CoA (mean, SD)	51.7 (8.7)	51.5 (7.7)	52.1 (8.2)	51.9 (9.5)	

SMMSE, Standardized Mini-Mental State Examination; CDR, cognitive drug research; SRT, simple reaction time; CRT, choice reaction time; DVT, digit vigilance task; PoA, power of attention. ^a^Untransformed scores; ^b^season-specific quartiles were derived for each season of blood draw and combined to create season-specific 25(OH)D groups (SQ1−SQ4); middle quartiles were collapsed (SQ2 and SQ3) and served as a referent; ^c^Kruskal–Wallis test for ordered and non-normally distributed continuous variables; ^d^RTV, reaction time variability, expressed as coefficient of variation; ^e^CoA, continuity of attention, expressed in CoA arbitrary units.

### 25(OH)D and prevalent global cognitive impairment (SMMSE)

Of 773 participants with baseline cognitive and 25(OH)D status used in the logistic regression models (Fig. S1), 212 (27.4%) were classified as impaired (SMMSE ≤ 25, 32,31). After adjustment for sex, education, health and lifestyle factors (model 3), participants in the lowest and highest season-specific 25(OH)D groups had increased odds of cognitive impairment compared with participants belonging to the middle group (odds ratio 1.66, 95% confidence interval 1.06–2.60, *P *=* *0.03, and 1.62, 1.02–2.59, *P *=* *0.04, respectively) (Table2). In the ‘restricted cohort’, only associations between the lowest 25(OH)D group and global cognitive functioning remained (1.73, 1.09–2.75, *P *=* *0.02).

**Table 2:** Association between season-specific 25(OH)D groups^a^ and odds of prevalent cognitive impairment^b^ (OR, 95% CI)

Season-specific 25(OH)D group	Entire cohort *n *=* *773						Participants not taking vitamin D supplements/medication *n* = 624					
	Model 1		Model 2		Model 3		Model 1		Model 2		Model 3	
	OR (95% CI)	*P*	OR (95% CI)	*P*	OR (95% CI)	*P*	OR (95% CI)	*P*	OR (95% CI)	*P*	OR (95% CI)	*P*
		<0.001		<0.001		0.04		<0.001		<0.001		0.06
Lowest	2.52 (1.71–3.70)	<0.001	2.42 (1.64–3.57)	<0.001	1.66 (1.06–2.60)	0.03	2.68 (1.80–4.00)	<0.001	2.56 (1.71–3.83)	<0.001	1.73 (1.09–2.75)	0.02
Middle	1		1		1		1		1		1	
Highest	2.11 (1.43–3.12)	<0.001	2.16 (1.45–3.21)	<0.001	1.62 (1.02–2.59)	0.04	1.32 (0.75–2.30)	0.34	1.31 (0.75–2.29)	0.35	1.45 (0.78–2.70)	0.24

OR, odds ratio; CI, confidence interval. Model 1 is unadjusted; model 2 is adjusted for sex and years of education; model 3 is additionally adjusted for number of income sources, smoking status, current alcohol consumption, waist−hip ratio, cardiovascular diseases (hypertension, cardiac disease, peripheral vascular diseases), cerebrovascular diseases, diabetes, osteoporosis, renal impairment, depressive symptoms and physical activity. The following missing variables were imputed to the reference value: education (*n *=* *11, ref. ≤9 years), number of income sources (*n *=* *5, ref. ≥2), smoking status (*n *=* *2, ref. never smoker), alcohol consumption (*n *=* *4, ref. no), renal impairment (*n *=* *1, ref. no), depressive symptoms (*n *=* *9, ref. no depressive symptoms) and physical activity (*n *=* *6, ref. low). ^a^Serum 25(OH)D was categorized in season-specific quartiles [lowest (SQ1), middle (SQ2 + SQ3) and highest (SQ4)]; middle quartiles were collapsed and served as the referent thus forming three season-specific groups; ^b^cognitive impairment was defined as scoring <26 points of the SMMSE.

### 25(OH)D and incident cognitive impairment over 3 years

Compared with participants who had SMMSE data 3 years later (*n *=* *470), those lost to follow-up [*n *=* *375 (44.4%); of whom 360 died and 15 did not complete the follow-up SMMSE] had fewer sources of retirement income (*P *=* *0.02), were less physically active (*P *<* *0.001), had more depressive symptoms (*P *=* *0.007) and chronic diseases (*P *=* *0.006), and were more likely to be cognitively impaired at baseline (*P *<* *0.001). Participants who remained in the study were more likely to take other vitamin supplements (*P *=* *0.001) but not prescribed vitamin D medication (*P *=* *0.001) and were more likely to drink alcohol (*P *<* *0.001). The groups did not differ on serum 25(OH)D status (*P *=* *0.94).

At the 3-year follow-up, 299 (66.2%) participants with 25(OH)D status were cognitively intact (SMMSE ≥ 26) and 153 (33.8%) were cognitively impaired (SMMSE ≤ 25) (Fig. S1). Of those cognitively intact at baseline, with established 25(OH)D status and with follow-up SMMSE (*n *=* *362), 82 (22.7%) decreased by ≥1 point and crossed the 25-point threshold. Similar models as for prevalent cognitive impairment were used to investigate the relationship between season-specific 25(OH)D groups and incident cognitive decline (Table3), but there was no evidence of a significant effect of 25(OH)D. These conclusions remained for fully adjusted models when the outcome was defined as global cognitive status 3 years later (Table S2), as a decline of ≥3 points of the SMMSE, as a decline of −1.0 SD or −1.5 SD below the mean using a 23-point cut-off to define cognitive impairment, as continuous outcome (i.e. difference scores), or when analyses were additionally controlled for vital status (dead or alive 2 years after the 3-year follow-up) to exclude possible terminal decline (data not shown).

**Table 3:** Association between season-specific 25(OH)D groups^a^ and odds of incident impairment^b^ 3 years later

Season-specific 25(OH)D group	Entire cohort, *n *=* *452						Participants not taking vitamin D supplements/medication, *n* = 380					
	Model 1		Model 2		Model 3		Model 1		Model 2		Model 3	
	OR (95% CI)	*P*	OR (95% CI)	*P*	OR (95% CI)	*P*	OR (95% CI)	*P*	OR (95% CI)	*P*	OR (95% CI)	*P*
		0.24		0.28		0.37		0.31		0.26		0.23
Lowest	0.98 (0.55–1.75)	0.94	0.97 (0.54–1.74)	0.92	1.03 (0.57–1.89)	0.91	1.10 (0.61–2.00)	0.94	1.13 (0.62–2.06)	0.70	1.26 (0.67–2.38)	0.48
Middle	1		1		1		1		1		1	
Highest	0.57 (0.30–1.11)	0.10	0.59 (0.30–1.15)	0.12	0.62 (0.31–1.25)	0.18	0.52 (0.21–1.30)	0.34	0.50 (0.20–1.26)	0.14	0.51 (0.20–1.31)	0.16

OR, odds ratio; CI, confidence interval. Model 1 is unadjusted; model 2 is adjusted for sex and years of education; model 3 is additionally adjusted for number of income sources, smoking status, current alcohol consumption, waist−hip ratio, cardiovascular diseases (hypertension, cardiac disease, peripheral vascular diseases), cerebrovascular diseases, diabetes, osteoporosis, renal impairment, depressive symptoms and physical activity. The following missing variables were imputed to the reference value: education (*n *=* *11, ref. ≤9 years), number of income sources (*n *=* *5, ref. ≥2), smoking status (*n *=* *2, ref. never smoker), alcohol consumption (*n *=* *4, ref. no), renal impairment (*n *=* *1, ref. no), depressive symptoms (*n *=* *9, ref. no depressive symptoms) and physical activity (*n *=* *6, ref. low). ^a^Serum 25(OH)D was categorized in season-specific quartiles [lowest (SQ1), middle (SQ2 + SQ3) and highest (SQ4)]; middle quartiles were collapsed and served as the referent thus forming three season-specific groups; ^b^cognitive impairment was defined as scoring <26 points of the SMMSE.

### 25(OH)D and attention (CDR attention battery)

The associations between season-specific 25(OH)D groups and attention reaction times and attention-specific composite scores at baseline and at 1.5- and 3-year follow-ups were examined through multilevel models. All attention-specific reaction times (including information processing speed) showed a significant increase over 3 years (i.e. slower or poorer performance) after adjustment for potential confounders (Table4). The linear growth rate for SRT, CRT, DVT and PoA reaction times increased (slowed) significantly by 0.028, 0.021, 0.009 and 0.023 log-transformed (mean) seconds per unit of time, respectively (Table4, model 3). CoA (sustained attention) declined over time (i.e. negative *β* estimates in models with untransformed scores; data not shown). No linear time trend level was evident for RTV, indicating little or no within-person change over 3 years.

**Table 4:** Parameter estimates^a^ of growth curve models for attention tasks over 3 years by season-specific 25(OH)D groups^b^ (entire cohort)

Outcome	Effects	Model 1 *β* (SE)	*P*	Model 2 *β* (SE)	*P*	Model 3 *β* (SE)	*P*
SRT	Time	0.029 (0.005)	<0.001	0.028 (0.007)	<0.001	0.028 (0.007)	<0.001
Intercept	25(OH)D						
	Lowest	0.031 (0.011)	0.006	0.030 (0.012)	0.009	0.02 (0.012)	0.09
	Middle (ref.)	0.0		0.0		0.0	
	Highest	0.025 (0.011)	0.03	0.025 (0.012)	0.03	0.023 (0.012)	0.05
Slope	25(OH)D × time	n/a					
	Lowest × time			0.004 (0.013)	0.75	0.004 (0.013)	0.74
	Middle × time (ref.)			0.0		0.0	
	Highest × time			−0.002 (0.013)	0.90	−0.002 (0.012)	0.85
CRT	Time	0.025 (0.004)	<0.001	0.021 (0.006)	<0.001	0.021 (0.006)	<0.001
Intercept	25(OH)D						
	Lowest	0.032 (0.009)	0.001	0.031 (0.009)	0.001	0.023 (0.009)	0.01
	Middle (ref.)	0.0		0.0		0.0	
	Highest	0.026 (0.009)	0.006	0.025 (0.009)	0.007	0.021 (0.009)	0.02
Slope	25(OH)D × time	n/a					
	Lowest × time			0.005 (0.010)	0.65	0.007 (0.010)	0.48
	Middle × time (ref.)			0.0		0.0	
	Highest × time			0.009 (0.010)	0.38	0.007 (0.012)	0.47
DVT	Time	0.079 (0.002)	<0.001	0.008 (0.003)	0.002	0.009 (0.004)	0.01
Intercept	25(OH)D						
	Lowest	0.014 (0.004)	0.002	0.013 (0.004)	0.004	0.009 (0.004)	0.05
	Middle (ref.)	0.0		0.0		0.0	
	Highest	0.011 (0.004)	0.006	0.012 (0.004)	0.004	0.010 (0.004)	0.02
Slope	25(OH)D × time	n/a					
	Lowest × time			0.002 (0.005)	0.61	0.002 (0.005)	0.66
	Middle × time (ref.)			0.0		0.0	
	Highest × time			−0.004 (0.005)	0.39	−0.004 (0.005)	0.34
PoA	Time	0.026 (0.003)	<0.001	0.023 (0.005)	<0.001	0.023 (0.004)	<0.001
Intercept	25(OH)D						
	Lowest	0.024 (0.007)	0.001	0.025 (0.007)	0.001	0.017 (0.007)	0.02
	Middle (ref.)	0.0		0.0		0.0	
	Highest	0.026 (0.007)	<0.001	0.026 (0.007)	<0.001	0.022 (0.007)	0.002
Slope	25(OH)D × time	n/a					
	Lowest × time			0.009 (0.008)	0.27	0.009 (0.008)	0.27
	Middle × time (ref.)			0.0		0.0	
	Highest × time			0.003 (0.008)	0.71	0.003 (0.008)	0.71
RTV	Time	0.003 (0.004)	0.43	0.004 (0.005)	0.46	0.005 (0.005)	0.38
Intercept	25(OH)D						
	Lowest	0.024 (0.008)	0.003	0.03 (0.009)	0.003	0.021 (0.009)	0.02
	Middle (ref.)	0.0		0.0		0.0	
	Highest	0.018 (0.008)	0.03	0.017 (0.009)	0.06	0.020 (0.009)	0.03
Slope	25(OH)D × time	n/a					
	Lowest × time			−0.007 (0.01)	0.48	−0.005 (0.01)	0.61
	Middle × time (ref.)			0.0		0.0	
	Highest × time			0.003 (0.009)	0.78	0.002 (0.009)	0.80
CoA	Time	0.091 (0.041)	0.03	0.107 (0.056)	0.06	0.122 (0.054)	0.03
Intercept	25(OH)D						
	Lowest	0.313 (0.087)	<0.001	0.325 (0.093)	0.001	0.294 (0.092)	0.001
	Middle (ref.)	0.0		0.0		0.0	
	Highest	0.029 (0.087)	0.74	0.039 (0.93)	0.68	0.134 (0.092)	0.14
Slope	25(OH)D × time	n/a					
	Lowest × time			−0.038 (0.104)	0.71	−0.030 (0.10)	0.77
	Middle × time (ref.)			0.0		0.0	
	Highest × time			−0.033 (0.102)	0.75	−0.040 (0.098)	0.68

SRT, simple reaction time; CRT, choice reaction time; DVT, digit vigilance task; PoA, power of attention; RTV, reaction time variability; CoA, continuity of attention. Model 1 includes serum 25(OH)D and linear trend of time; in model 2 a linear trend of time by serum 25(OH)D interaction is added; model 3 is further adjusted for education, sex, smoking status, current alcohol intake, renal impairment and number of chronic diseases (0–1 diseases, 0; two diseases, 1; three and more diseases, 2). ^a^Estimated *β* values (SE) of fixed effects using transformed longitudinal data for all outcomes. Random effects terms included both intercept and slopes of attention scores over time. Time in the study was coded as baseline (0), 1.5-year follow-up (1) and 3-year follow-up (2). ^b^Serum 25(OH)D was categorized in season-specific quartiles. Middle quartiles (SQ2 and SQ3) were combined and served as the reference group.

Initial status (intercept) for all attention outcomes varied significantly by season-specific 25(OH)D quartiles (model 1) but the rate of change in attention (slope) showed no effect (models 2 and 3). Specifically, after adjustment for sex, education, lifestyle factors and number of chronic conditions (model 3), both the lowest and highest quartiles of 25(OH)D were associated with overall slower reaction times and information processing speed in CRT and DVT and with increased PoA (i.e. focused attention/intensity of concentration) and RTV scores (i.e. greater fluctuation in attention) compared with the middle quartile. The log-transformed means of DVT sustained attention speed were slower by 0.009 s (*P *=* *0.05) and by 0.010 s (*P *=* *0.002) for participants belonging to the lowest and highest 25(OH)D quartiles, respectively. The log-transformed RTV coefficients of variance were higher by 0.021 (*P *=* *0.02) for those in the lowest quartile and by 0.020 (*P *=* *0.03) for participants in the highest 25(OH)D quartile, indicating greater fluctuation in attention compared with participants in the middle 25(OH)D group. A significant effect of highest 25(OH)D quartile on PoA [*β* (SE) = 0.022 (0.007), *P *=* *0.002] but not on CoA (*P *=* *0.14) was also observed, suggesting that the overall change in PoA speed amongst these participants was independent of the poorer ability to sustain attention/accuracy. A non-significant time by 25(OH)D group interaction for all outcomes and models indicated that the slopes (rate of change) did not vary by 25(OH)D quartile between individuals over the study period (Table4). In the ‘restricted cohort’, similar conclusions regarding 25(OH)D and attention-specific outcomes held for the lowest but not the highest 25(OH)D group (Table S3).

## Discussion

In this prospective, population-based study of older adults aged 85+, it was found that both low and high season-specific quartiles of 25(OH)D were associated with higher odds of prevalent cognitive impairment (assessed by SMMSE), poorer attention reaction times/processing speed and focused attention/concentration, and greater attention fluctuation (assessed by CDR). Differences remained significant after adjustment for sex, education, lifestyle factors and the presence of several chronic diseases, although effects were small. In the fully adjusted model the harmful effect of the highest season-specific 25(OH)D quartile on focused attention (PoA) seemed to be independent of the ability to sustain attention/accuracy (CoA), suggesting no concentration−accuracy trade-offs. However, the rate of change of all attention measures did not vary across 25(OH)D groups, and no association between 25(OH)D and odds of global incident cognitive impairment or decline was found. In analyses restricted to vitamin D supplements/medication non-users, only the associations with the lowest season-specific 25(OH)D group remained for both global cognitive and attention-specific outcomes.

To our knowledge, this is the first prospective study to find evidence for a U-shaped relationship between 25(OH)D and global cognitive function and attention in the very old. Taken together, it could be hypothesized that the neuroprotective effects of vitamin D mediated via expression of proteins that, for example, attenuate the toxicity of reactive oxygen species 44 in very old neurons are attained only at moderate but not at low or high 25(OH)D concentrations.

Thus far, only four prospective studies of older adults aged 65+ have examined the association between 25(OH)D and prevalent and incident global cognitive impairment, and decline in attention and executive function, with inconsistent results. A study of community-dwelling older men 22 found limited evidence of an independent association between lower 25(OH)D concentration (≤19.9 ng/ml) and incident cognitive impairment or decline in global and executive function. A similar study involving community-dwelling older women 23 reported that very low (<25 nM) and low levels (<50 nM) of 25(OH)D were associated with an increased risk of impaired global cognitive function and decline [defined by modified MMSE (3MS)], but not with impaired executive function or decline. Two further studies have also investigated these 25(OH)D cut-points. The InCHIANTI study found that compared with participants with sufficient 25(OH)D (≥75 nM) the deficient group (<25 nM) experienced a substantial global (assessed by MMSE) and executive cognitive decline (assessed by Trails A and B) over 6 years 24, whilst the Health, Aging and Body Composition Study confirmed that lower 25(OH)D (<50 nM) was associated with a greater cognitive decline on the 3MS compared with sufficient 25(OH)D (≥75 nM) over 4-year follow-up 25.

A similar global cognitive measure as in previous studies was utilized, although different serum 25(OH)D cut-offs were derived *a posteriori* 42, but no association between 25(OH)D and global incident impairment or decline after adjustment for confounders was detected. This lack of association may be due to the age of our participants, reduced power to detect the association, specific definition of cognitive change at an individual level, and/or changed serum 25(OH)D status over the 3 years of the study. Increased mortality amongst older women belonging to the lowest and highest season-specific 25(OH)D quartiles as observed in this cohort 45 could be one of the reasons for the loss of analytical power. Incident impairment was defined as crossing the 25-point threshold of SMMSE 32,31. Although only five participants converted from cognitively normal to impaired by losing one point, a loss of <3 points may not represent a true change at an individual level.

Reported adverse effects of higher 25(OH)D appeared to be driven by those taking prescribed vitamin D medication [*n *=* *139 (16.5%)],who may have had prior (long-standing) vitamin D deficiency. Therefore, a potential negative effect of the highest 25(OH)D quartile on cognitive functioning could be partly driven by those with chronic vitamin D inadequacy who through supplementation reached higher concentrations shortly before baseline assessments. Nonetheless our finding is in agreement with reports from the NHANES III, a cross-sectional study of the non-institutionalized US population, aged 60–90 years, where the worst performance on learning and memory tasks was associated with the highest quintile of 25(OH)D 21. The Institute of Medicine Committee (2011) 7 emphasized a curvilinear association between 25(OH)D and non-skeletal health outcomes (e.g. all-cause mortality, cancers, cardiovascular diseases) and cautioned against the assumption that higher 25(OH)D concentrations (>50 nM) inevitably provide greater health benefits. This has been confirmed by a large retrospective study that included older adults aged 75+ from Copenhagen general practices, which found the lowest risk of all-cause mortality over 3 years was associated with 50–60 nM of 25(OH)D 6.

About 27% of participants scored <26 SMMSE points at baseline. There is therefore the possibility of reverse causation (i.e. non-optimal 25(OH)D concentrations being a consequence of prevalent cognitive impairment) 46, although excluding those with dementia diagnosis in sensitivity analyses (*n *=* *57) did not change the U-shaped association between vitamin D groups and baseline cognitive impairment after adjusting for covariates (lowest, odds ratio 1.89, 95% confidence interval 1.18–3.03, *P *=* *0.008; highest, 1.82, 1.11–2.97, *P *=* *0.02; data not shown].

To assess change in attention/information processing speed, attention fluctuation and accuracy in relation to 25(OH)D, the CDR system, previously used in dementia studies and clinical trials to discriminate between various types of dementias and to detect change in attention-specific cognitive domains pre and post treatment with millisecond precision, was employed 36,37,47. Specifically, focused attention (PoA) showed a clinically meaningful decline of 59 ms over 6 months in very mildly impaired Alzheimer's disease patients (MMSE >26) taking cholinesterase inhibitors 48. In the present study, participants in the lowest and highest season-specific 25(OH)D quartiles had overall worse focused attention and were by ∽96 ms and ∽86 ms slower (mean of raw scores), respectively, than those in the middle group at 18 months’ follow-up. Future studies will determine whether these attention deficits relate to decline in global cognition and interfere with daily functioning 49.

A small clinical trial of patients aged 65 and over with a history of falls and 25(OH)D insufficiency (≤12 ng/ml) showed an improvement of 0.4 s in CRT, compared with the control group, 6 months after a single intramuscular injection of vitamin D, which increased the average serum 25(OH)D from 10.4 to 17.5 ng/ml – the latter within the middle quartiles reported here to be associated with better attention scores 50.

There are some limitations of our study mainly related to loss to follow-up, use of a single measure of 25(OH)D and unknown 25(OH)D status prior to baseline assessments. Although participants remaining in the study had similar 25(OH)D concentrations to those lost to follow-up, they were less cognitively impaired and depressed, had fewer chronic diseases and were less likely to take prescribed vitamin D medication. In this cohort, prescribed vitamin D was a significant determinant of the highest, and less beneficial, serum 25(OH)D concentration. A single measurement of circulating 25(OH)D may inadequately reflect overall vitamin D status due to its cyclical nature 41, although a preferred statistical method was used to adjust for seasonal variability in 25(OH)D when only one sample was available 42. Generalization of our findings may be limited to adults aged 85+ of white ethnicity living at similar latitudes (55°N). Lastly, observed statistically significant worse attention scores have limited interpretability and may not be clinically meaningful and warrant treatment or intervention.

The strengths of our study include (i) its prospective design, the representativeness of the cohort and inclusion of the institutionalized older adults who were assessed at residency to reduce attrition and selection bias 28,29; (ii) the inclusion of several cognition-related covariates in multivariate analyses with the entire cohort and ‘restricted cohort’; and (iii) implementation of global and attention-specific tests that were validated and pilot-tested in this age group 40.

In summary, it was observed that both low and high season-specific concentrations of 25(OH)D were associated with increased risk of prevalent cognitive impairment and worse attention-specific tasks amongst older adults aged 85+, although the rates of change were similar across vitamin D groups. Poorer cognitive functioning in participants belonging to the highest season-specific 25(OH)D group was driven by those receiving vitamin D supplements/medication. Future prospective studies should test the proposed U-shaped relationship between serum 25(OH)D and cognition in this age group and determine whether other cognitive domains are affected similarly by 25(OH)D status.

## Supporting Information

**Method S1.**Cognitive assessment. **Method S2.** Serum 25(OH)D assay. **Method S3.** Season-specific 25(OH)D quartile cut-offs. **Method S4.** Description and coding of confounders used in logistic regression and growth models. **Method S5.** Statistical analysis. **Figure S1.**Newcastle 85+ Study participants by SMMSE scores and 25(OH)D status availability over the study period. **Table S1.** Baseline characteristics of participants in the Newcastle 85+ Study by serum 25(OH)D. **Table S2.** Association between season-specific 25(OH)D groups and odds of cognitive impairment (odds ratio, 95% confidence interval) at 3-year follow-up. **Table S3.** Parameter estimates of growth curve models for attention tasks over 3 years by season-specific 25(OH)D groups (‘restricted cohort’).
