# Supplementary material for: Serum 25-hydroxyvitamin D and cognitive decline in the very old: the Newcastle 85+ Study
Source: Eur J Neurol. 2014 Aug 13;22(1):106–e7. doi: 10.1111/ene.12539 (PMC4310141; doi:10.1111/ene.12539)
Supplement: Supplementary file 1 — Method S1.Cognitive assessment. Method S2. Serum 25(OH)D assay. Method S3. Season-specific 25(OH)D quartile cut-offs. Method S4. Description and coding of confounders used in logistic regression and growth models. Method S5. Statistical analysis. Figure S1.Newcastle 85+ Study participants by SMMSE scores and 25(OH)D status availability over the study period. Table S1. Baseline characteristics of participants in the Newcastle 85+ Study by serum 25(OH)D. Table S2. Association between season-specific 25(OH)D groups and odds of cognitive impairment (odds ratio, 95% confidence interval) at 3-year follow-up. Table S3. Parameter estimates of growth curve models for attention tasks over 3 years by season-specific 25(OH)D groups (‘restricted cohort’). [file ene0022-0106-sd1.docx]

**Supplementary Information**

**Supplementary Method (5)**

Supplementary Method 1: Cognitive assessment

Supplementary Method 2: Serum 25(OH)D assay

Supplementary Method 3: Season-specific 25(OH)D quartiles cut-offs

Supplementary Method 4: Description and coding of confounders used in logistic regression and growth models.

Supplementary Method 5: Statistical analysis

**Supplementary References:** 1-4

**Supplementary Figure Legend: Supplementary Figure 1**

**Supplementary Tables (3)**

Supplementary Table 1: Baseline characteristics of participants in the Newcastle 85+ study by serum 25(OH)D

Supplementary Table 2: Association between season-specific 25(OH)D groups and odds of cognitive impairment (OR, 95% CI) at 3-year follow-up

Supplementary Table 3: Parameter estimates of growth curve models for attention tasks over 3 years by season-specific 25(OH)D groups (‘restricted cohort’)

**Supplementary Figure**

**Supplementary Figure 1:** Newcastle 85+ Study participants by SMMSE scores and 25(OH)D status availability over the study period.

**Supplementary Methods**

**Supplementary Method 1**

**Cognitive assessments**

**Administration of CDR attention battery**

The CDR tasks were presented on a hi-resolution Windows-based laptop computer (Motion Computing® LE1600 Tablet PC with keyboard accessory), and participants responded using a two-button (YES/NO) response box. The assessment battery took approximately 15 minutes to complete. A training session was performed to familiarize participants with the computerized testing procedures, approximately one week before collection of the assessment data, using the same tasks but with fewer stimuli. For a given participant, the same research nurse administered all cognitive tasks and gave standardized task instructions verbally. Where it was obvious the participant had not understood the task correctly, the instructions were repeated and/or reworded. Tasks could be paused, re-started or repeated if it was clear from the participant or their performance that they had misunderstood the nature of the task. However, tasks were not re-started or repeated if the participant had understood the instructions but performance was poor. If a participant was agitated by the testing e.g. by being unable to understand the task despite repeated explanations or distressed by their performance, the task could be omitted or aborted at the discretion of the research nurse. An electronic log was kept for each session documenting task completion and the reason for any missing data.

**Description of the CDR attention battery tasks and measures**

Six attention-specific measures/tasks were used as main outcomes in relation to serum 25(OH)D as follows:

1. **Simple Reaction Time (SRT)**: The participant is instructed to press the ‘YES’ response button as quickly as possible every time the word ‘YES’ is presented on the screen. Thirty ‘YES’ only stimuli are presented, with a varying inter-stimulus interval. Mean reaction times (standard deviation) are calculated in milliseconds (ms). Lower scores represent better performance.

2. **Choice Reaction Time (CRT)**: Either the word ‘YES’ or ‘NO’ is presented on the screen, and the participant is instructed to press corresponding button as quickly as possible. There 30 trials for which each stimulus word is chosen randomly with equal probability, with a varying inter-stimulus interval. Mean reaction times (standard deviation), are calculated in milliseconds (ms). Lower scores represent better performance. Percentage (%) of accurate responses are also recorded.

3. **Digit Vigilance Task (DVT)**: A target digit is randomly and constantly displayed to the right of the screen. A series of digits (0-9) is presented in the center of the screen at the rate of 150 per minute. The participant is required to press the ‘YES’ button as quickly as possible every time the digit in the series matches the target digit. There are 300 digits in the series and the task lasts 2 minutes. Mean reaction times (standard deviation), are calculated in milliseconds (ms). Lower scores represent better performance. Percentage (%) of accurate responses and false alarms (number of responses to non-targets) are also recorded.

4. **Power of Attention (PoA)**: A composite score calculated by summing SRT, CRT and DVT mean reaction times (ms). Measures intensity of concentration: the faster the response, the more resources are being brought to bear upon task. Lower scores represent better performance.

5. **Reaction Time Variability (RTV)**: A composite score calculated by summing up the coefficients of variance from SRT, CRT and DVT mean reaction times. Measures fluctuation in attention and consistency in responding to correct target stimuli. Lower scores (coefficient of variance) represent better performance.

6. **Continuity of Attention (CoA)**: A composite score calculated by combining the accuracy scores from the CDR and DVT (CRT accurate responses*0.30 + DVT accurate responses*0.30 –DVT false alarms). Measures ability to sustain attention. Higher scores represent better performance.

**Composite scores construction and validation**

PoA and CoA were originally identified by a principal components analysis (PCA) from a large cohort of middle aged volunteers [37]. Briefly, all measures from the entire CDR assessment system were submitted to PCA to determine the factor structure of the various measures. The scores from tests of working memory (articulatory and spatial) and episodic memory (verbal and non-verbal) were also included in the analysis. A clear factor structure emerged: two of the factors involving all six attention task measures, and three other factors on which the measures from the working and episodic memory tasks loaded independently. The three speed scores form the attention tasks compromised one discrete factor termed Power of Attention (PoA). The accuracy scores from the attention tasks comprised another factor termed Continuity of Attention (CoA). The PCA demonstrated that the CDR factors are relatively independent i.e. that the speed of attentional processes is free to vary independent of the accuracy of attention, and that attentional processes are relatively independent of those associated with memory.

Subsequent work evaluated measures of the variability of the reaction times in the three tasks (i.e. SRT, CRT and DVT) [38] based on the standard deviation of individual responses. This work showed that variability was an independent measure of attentional performance which tied closely to clinical measures of attentional fluctuations. Reaction Time Variability (RTV) is a score derived from the coefficients of variation of the reaction times from the three tasks, independent of the overall mean of the reaction times.

The composite attention measures have been successfully used to profile the effects of various compounds on cognition (e.g. the effects of rivastigmine in patients with Parkinson’s Disease Dementia [39]).

**Supplementary Method 2**

**Serum 25(OH)D assay**

Fasting blood samples were drawn between 7 am and 10:30 am and delivered to the Royal Victoria Infirmary (Newcastle upon Tyne, UK) laboratory for processing within one hour of draw. Serum 25-hydroxyvitamin D (25(OH)D) was measured by DiaSorin Radioimmune Assay (RIA ) kit (DiaSorin Corporation, Stillwater, MN ), a two-step procedure for a rapid extraction of 25-hydroxy-ergocalciferol (25(OH)D_2_), 25-hydroxy-cholecalciferol (25(OH)D_3_) and other hydroxylated metabolites. In the first step, two 50 μL blood serum aliquots of each sample were subjected to acetonitrile precipitation. In the second step, after extraction, the samples were assayed by RIA method using 25(OH)D-specific antibodies and ^125^I-labelled 25(OH)D (Diasorin Corporation) as a tracer. The sample, antibodies and tracer were coincubated for 90 min at 20-25º C, following phase separation with secondary antibody-precipitating complex for additional 20 min incubation at 20-25º C. Next, a NSB/Addition Buffer was added prior to centrifugation to minimize non-specific binding. The samples were measured using a Packard COBRA Quantum Gamma Counter (Packard Instrument Company, Downers Grove, IL) and calculations accomplished with the accompanied software. The minimum detectable concentration of 25(OH)D was 6 nmol/L, and inter-assay coefficients of variation were 8.4 to 12.6% (working range of assay: 6-250 nmol/L).

**Supplementary Method 3**

**Season-specific serum 25(OH)D quartiles cut-offs**

Season-specific serum 25(OH)D quartiles (SQ1 to SQ4) were created for each season of blood collection to account for seasonal variation of UVB exposure and vitamin D skin production [42]. Serum 25(OH)D quartiles cut-offs for each seasons were as follows.

*Summer* (June-August): SQ1 (5-28 nmol/L), SQ2 (29-45 nmol/L), SQ3 (46-68 nmol/L) and SQ4 (≥69 nmol/L).

*Autumn* (September-November): SQ1 (8-30 nmol/L), SQ2 (31-43 nmol/L), SQ3 (44-61 nmol/L), and SQ4 (≥62 nmol/L).

*Winter* (December-February): SQ1 (6-22 nmol/L), SQ2 (23-32 nmol/L), SQ3 (33-59 nmol/L), and SQ4 (≥60 nmol/L).

*Spring* (March-May): SQ1 (5-17 nmol/L), SQ2 (18-26 nmol/L), SQ3 (27-46 nmol/L), and SQ4 (≥47 nmol/L).

**Supplementary Method 4**

**Description and coding of confounders used in logistic regression and growth models**

**Sociodemographic variables***:* (a) gender (categorical): women/men (0/1); (b) years of education (ordered): 0-9/10-11/≥12 years; (c) sources of income (ordered): 2/3/4-5 sources which include state retirement pension, occupational pension, private pension, savings/investments, and welfare benefits.

**Heath and morbidity-related variables***:* (a) waist-hip ratio (continuous): calculated as waist (cm)/hip (cm) and imputed with sex-specific means for 97 individuals with missing values; (b) number of chronic diseases (continuous), from the list: arthritis (e.g. generalized osteoarthritis, rheumatoid osteoarthritis, spondylosis, etc.), hypertension, cardiac disease (e.g. heart failure, angina, myocardial infarction, coronary angioplasty or stent, coronary artery bypass graft), respiratory disease (e.g. bronchiectasis, pulmonary fibrosis, asthma, chronic bronchitis, COPD, emphysema, etc.), cerebrovascular disease (e.g. stroke, transient ischaemic attack, carotid endarterectomy, etc.), diabetes (Type 1, Type 2, and unspecified), cancer (any cancer diagnosis in the past 5 years excluding non-

melanoma skin cancer); (c) renal impairment (binary): yes/no (1/0), the diagnosis determined by the Chronic Kidney Disease Epidemiology Collaboration (CKD-EPI) guidelines [1]; (d) serum vitamin B_12_ (ordinal): categorized in quartiles (cut-off points: 230, 314, 441ng/L; range 60-2000ng/L; middle quartiles collapsed to form a referent); (e) serum folate (ordinal): categorized in quartiles (cut-off points: 271, 383, 566µg/L; range 78-2000µg/L; middle quartiles collapsed to form a referent).

**Lifestyle-related variables***:* (a) physical activity (ordinal): low/moderate/high (0/1/2); (b) smoking status (categorical): never smoker/ former smoker/ current smoker (0/1/2); (c) current alcohol intake (binary): yes/no (1/0).

**Mental health-related variable***:* Geriatric Depression Scale (GDS [2]) (ordinal): scores 0-5 (no depressive symptoms), 6-7 (mild depression), ≥8 (severe depression); 45 participants (5.5% of total sample) who had a baseline SMMSE <15 did not complete GDS and were included as a separate category at baseline and follow-up for analyses of global cognition.

**Serum 25(OH)D-related variables***:* (a) vitamin D-containing supplements (categorical): yes, at least one/no but taking others/not taking any vitamin supplements (0/1/2); ‘taking others’ included non-prescribed multivitamins, multivitamins with minerals, and combination of vitamins A and C with other vitamins except D; (b) prescribed vitamin D medication (binary): yes/no (1/0), ‘yes’ included prescription vitamin D, calcium with vitamin D, bisphosphonate with calcium and vitamin D, and strontium with calcium and vitamin D.

**Supplementary Methods 5**

**Statistical analysis**

All analyses were performed using IBM SPSS Statistics software version 19 (IBM, New York).

**Characteristics of the participants lost to follow-up**

We compared participants lost to follow-up to those still in the study 3 years post baseline by Mann-Whitney U tests for ordered and non-normally distributed continuous data, and χ^2^ tests for categorical data.

**Multicollinearity of confounders**

Multicollinearity of confounders was assessed by examining the correlational matrix and multi-collinearity diagnostics (i.e. VIF, Tolerance, Eigenvalues and Condition Index). Multivariate outliers were tested with Mahalanobis’ distances.

**Sensitivity analysis**

Several sensitivity analyses were performed to investigate the association between season-specific 25(OH)D groups and cognitive function over the study period: (a) logistic regression models fitted to entire and ‘restricted cohort’ (excluding those on vitamin D supplements/medication) with non-imputed covariates and prevalent and incident impairment as outcomes; (b) models with cognitive impairment at 3-year follow-up as an outcome (Supplementary Table 2); (c) models in which cognitive decline was defined as loss of ≥3 SMMSE points or established by a Reliable Change Index [3] to account for measurement error and practice effect in SMMSE; (d) models in which cognitive decline was defined as -1 SD and -1.5 SD below the mean of SMMSE; (e) models in which cognitive impairment was defined as scoring 23 or less SMMSE points at baseline and 3-year follow-up; (f) additional models with serum vitamin B_12_ and folate as covariates (categorized in quartiles and combined middle quartiles as a referent); (g) models with a ‘vital status’ variable (i.e. alive or dead 2 years [26.37±4.02 months] after the 3-year follow-up) to control for terminal drop affecting the rate of incident cognitive impairment [4]; and (h) multivariable regression with SMMSE difference scores as continuous outcome, (g) aforementioned models fitted to participants without dementia/Alzheimer’s disease diagnosis (from GP records) at baseline (analytic sample of 716 participants); and (i) random effects models fitted to ‘restricted cohort’ with attention scores as outcomes (Supplementary Table 3). The -2 Restricted Log Likelihood and Akaike’s Information Criterion were used to determine the improvements of the goodness of fit in the models.

**Supplementary References**

1. [Stevens LA](http://www.ncbi.nlm.nih.gov/pubmed?term=Stevens%20LA%5BAuthor%5D&cauthor=true&cauthor_uid=17591522), [Manzi J](http://www.ncbi.nlm.nih.gov/pubmed?term=Manzi%20J%5BAuthor%5D&cauthor=true&cauthor_uid=17591522), [Levey AS](http://www.ncbi.nlm.nih.gov/pubmed?term=Levey%20AS%5BAuthor%5D&cauthor=true&cauthor_uid=17591522), *et al*. Impact of creatinine calibration on performance of GFR estimating equations in a pooled individual patient database. [*Am J Kidney Dis*](http://www.ncbi.nlm.nih.gov/pubmed/17591522) 2007; **50**: 21-35.

2. Yasavage JA. Geriatric Depression Scale. *Psychopharmacol Bull* 1988; **24**: 709-711.

3. Chelune GJ, Naugle RI, Lüders H, Sedlak J, Awar IA. Individual change after epilepsy surgery: Practice effects and base-rate information. *Neuropsychology.* 1993; **7**: 41-52.

4. Wilson RS, Beck TL, Bienias JL, [Bennett DA](http://www.ncbi.nlm.nih.gov/pubmed?term=Bennett%20DA%5BAuthor%5D&cauthor=true&cauthor_uid=17327212).Terminal cognitive decline: accelerated loss of cognition in the last years of life. *Psychosom Med* 2007; **69**: 131-137.

**Supplementary Figure Legends**

**Supplementary Figure 1**

**Newcastle 85+ Study participants by SMMSE scores and 25(OH)D status availability over the study period.**

Of 845 participants in the study, 773 (91.5%) had established 25(OH)D and cognitive status at baseline of which 561 (72.6%) were cognitively normal (scoring ≥26 points of the SMMSE), and 212 (27.4%) were impaired (scoring <26 SMMSE points). Over the study period, 375 (44.4%) participants were lost to follow-up. At 3-year follow-up, 452 had SMMSE scores and 25(OH)D status and were considered for prospective analysis.

Supplementary Table 1 Baseline characteristics of participants in the Newcastle 85+ study by season-specific 25(OH)D groups^a^

Characteristic All participants Lowest season-specific Middle 25(OH)D season-specific^b^  Highest season-specific

25(OH)D group 25(OH)D group 25(OH)D group

*n*=845 *n*=191 *n*=392 *n*=192 *P*^c^

Women % (n) 62.2 (526) 62.3 (119) 54.8 (215) 71.4 (137)^d^ 0.001

Years of education % (*n*)

0-9 y 64.4 (534) 68.3 (127) 62.4 (242) 63.2 (120)

10-11 y 22.8 (186) 25.8 (48) 23.2 (90) 22.1 (42)

≥12 y 12.8 (106) 5.9 (11) 14.4 (56) 14.7 (28)

Number of income sources *%* (*n*)

0, 1 or 2 30.3 (254) 36.8 (70) 24.4 (95) 33.2 (63)

3 46.7 (391) 41.6 (79) 52.1 (203) 43.2 (82)

4 or 5 23.0 (193) 21.6 (41) 23.6 (92) 23.7 (45)

Vitamin D supplements % (*n*) <0.001

Yes 3.8 (32) 1.0 (2) 3.8 (15) 6.3 (12)

No but taking other supplements 37.4 (316) 26.2 (50) 42.1 (165) 44.8 (86)

Not taking any supplements 58.8 (497) 72.8 (139)^d^  54.1 (212) 49.0 (94)

Prescribed medication w. vitamin D % (*n*) <0.001

Yes 16.5 (139) 0.5 (1) 7.9 (31) 47.9 (92)^d^

Waist-hip ratio (mean, SD) 0.88 (0.07) 0.88 (0.07) 0.88 (0.07) 0.88 (0.08)

Serum B_12_ % (*n*)

Lowes level 25.2 (190) 29.4 (55) 25.0 (96) 21.4 (39)

Middle 49.8 (375) 46.5 (87) 51.3 (197) 50.0 (91)

Highest 25.0 (188) 24.1 (45) 23.7 (91) 28.6 (52)

Serum folate % (*n*)

Lowest level 24.7 (186) 33.2 (62) 22.2 (85) 21.4 (39) 0.006

Middle 50.2 (377) 47.6 (89) 51.4 (197) 50.0 (91)

Highest 25.1 (189) 19.3 (36) 26.4 (101) 28.6 (52)

Physical activity % (*n*)

Low 23.3 (189) 28.7 (54) 15.4 (60) 29.2 (56) <0.001

Moderate 43.0 (349) 46.8 (88) 42.7 (166) 39.6 (79)

High 33.7 (274) 24.5 (46) 41.9 (163) 31.3 (60)

Smoking status *%* (*n*)

Never 35.8 (301) 38.2 (73) 30.4 (119) 41.7 (80)

Current smoker 5.7 (48) 6.8 (13) 5.9 (23) 4.2 (8)

Former smoker 58.5 (491) 55.0 (105) 63.6 (248) 54.2 (104)

Current alcohol intake % (n)

Yes 59.5 (488) 51.6 (98)^d^ 65.6 (255) 59.9 (115) 0.005

Number of chronic diseases (mean, SD) 2.26 (1.22) 2.37 (1.25) 2.22 (1.21) 2.24 (1.25)

Cardiovascular diseases % (*n*)

Yes 72.0 (608) 80.4 (148) 74.2 (287) 66.8 (125)^d^  0.01

Cerebrovascular disease % (*n*)

Yes 21.1 (178) 22.0 (42) 19.9.8 (78) 23.4 (45)

Diabetes % (*n*)

Yes 13.3 (112) 16.8 (32) 14.3 (56) 10.4 (20)

Supplementary Table 1 (continued)

Characteristic All participants Lowest season-specific Middle season-specific^b^  Highest season-specific 25(OH)D group 25(OH)D group 25(OH)D group

*n*=845 *n*=191 *n*=392 *n*=192 *P*^c^

Osteoporosis % (*n*)

Yes 13.3 (112) 6.3 (12) 7.7 (30) 30.2 (58)^c^ <0.001

Dementia % (n)

Yes 8.8 (74) 8.4 (16) 6.4 (25) 9.4 (18)

Renal impairment % (n)

Yes 23.9 (185) 21.5 (41) 23.8 (93) 26.6 (51)

Depressive symptoms % (n)^e^

0-5/none 74.6 (607) 69.9 (131) 81.1 (314) 70.9 (134) <0.001

6-7/mild 12.0 (98) 12.1 (23) 11.6 (45) 12.7 (24)

≥8/severe 7.9 (64) 11.1 (21) 5.9 (23) 9.5 (18)

SMMSE <15 5.5 (45) 7.9 (15) 1.3 (5) 6.9 (13)

25(OH)D, 25-hydroxyvitamin D; SMMSE, Standardized Mini-Mental State Examination.

^a^Serum 25(OH)D was categorized in season-specific quartiles (lowest [SQ1], middle [SQ2+SQ3], and highest [SQ4]).^b^Middle season-specific quartiles of serum 25(OH)D (SQ2 and SQ3) were combined thus forming three season-specific groups. ^c^Kruskal-Wallis test for ordered and non-normally distributed continuous variables and χ^2^ test for categorical variables. ^d^In the *post hoc* χ^2^ test analyses, adjusted residuals were used to determine which cells were major contributors to rejecting the null hypothesis at α=0.05. ^e^Fifteen point Geriatric Depression Scale (GDS).

Supplementary Table 2 Association between season-specific 25(OH)D groups^a^ and odds of cognitive impairment^b^ (OR, 95% CI) at 3-year follow-up

**____________________________________________________________________________________________________________________**

Entire cohort  *Participants not taking vitamin D supplements/medication*

*n*=452 *n=380*

___________________________________________________________________________________________________________________________________________

Season-specific Model 1 Model 2 Model 3 *Model 1 Model 2 Model 3*

25(OH)D OR (95% CI) *P* OR (95% CI) *P* OR (95% CI) *P* *OR (95% CI)* *P* *OR (95% CI)* *P* *OR (95% CI)* *P*

group ___________________________________________________________________________________________________________________________________________

0.13 0.14 0.30 0.02 0.02 0.04

Lowest 1.60 (0.93-2.75) 0.09 1.55 (0.90-2.69) 0.12 1.43 (0.81-2.51) 0.22 1.83 (1.06-3.16) 0.03 1.81 (1.04-3.14) 0.04 1.73 (0.96-3.10) 0.07

Middle 1 1 1 1 1 1

Highest 0.85 (0.48-1.50) 0.60 0.83 (0.47-1.47) 0.52 0.83 (0.44-1.54) 0.55 0.57 (0.26-1.26) 0.17 0.55 (0.25-1.23) 0.15 0.56 (0.25-1.29) 0.17

___________________________________________________________________________________________________________________________________________

^a^Serum 25(OH)D was categorized in season-specific quartiles. Middle quartiles were collapsed and served as the referent. ^b^Cognitive impairment was defined as scoring less than 26 points of the SMMSE.

Model 1 is adjusted for cognitive status at baseline.

Model 2 is additionally adjusted for sex and years of education.

Model 3 is additionally adjusted for number of income sources, smoking status, and current alcohol consumption, waist-hip ratio, cardiovascular diseases (hypertension, cardiac disease, peripheral vascular diseases), cerebrovascular diseases, diabetes, osteoporosis, renal impairment, depressive symptoms, and physical activity.

The following missing variables were imputed to the reference value: education (*n*=11, ref: ≤9 years), number of income sources (*n*=5, ref: ≥2), smoking status (*n*=2, ref: never smoker), alcohol consumption (*n*=4, ref: no), renal impairment (*n*=1, ref: no), depressive symptoms (*n*=9, ref: no depressive symptoms), and physical activity (*n*=6, ref: low).

Supplementary Table 3 Parameter estimates^a^ of growth curve models for attention tasks over 3 year by season-specific 25(OH)D groups^b^ (‘restricted cohort’) ___________________________________________________________________________________________________________________

Outcome Effects Model 1 *P* Model 2 *P* Model 3 *P*

β (SE) β (SE) β (SE)

_____________________________________________________________________________________________________________________________

SRT Time 0.029 (0.005) <0.001 0.030 (0.007) <0.001 0.030 (0.007) <0.001

Intercept 25(OH)D

Lowest 0.040 (0.011) <0.001 0.039 (0.011) <0.001 0.031 (0.011) 0.006

Middle (ref) 0.0 0.0 0.0

Highest -0.004 (0.014) 0.78 -0.002 (0.014) 0.89 -0.002 (0.014) 0.87

Slope 25(OH)D X Time n/a

Lowest X Time 0.004 (0.012) 0.76 0.004 (0.012) 0.72

Middle X Time (ref) 0.0 0.0

Highest -0.010 (0.015) 0.49 -0.010 (0.015) 0.48

__________________________________________________________________________________________________________________

CRT Time 0.022 (0.005) <0.001 0.021 (0.006) 0.001 0.021 (0.006) <0.001

Intercept 25(OH)D

Lowest 0.037 (0.009) <0.001 0.036 (0.009) <0.001 0.028 (0.009) 0.003

Middle (ref) 0.0 0.0 0.0

Highest -0.005 (0.012) 0.64 -0.005 (0.012) 0.67 -0.008 (0.012) 0.52

Slope 25(OH)D X Time n/a

Lowest X Time 0.007 (0.011) 0.49 0.008 (0.010) 0.47

Middle X Time (ref) 0.0 0.0

Highest -0.002 (0.013) 0.86 -0.002 (0.013) 0.85

__________________________________________________________________________________________________________________

DVT Time 0.009 (0.002) <0.001 0.009 (0.003) 0.001 0.009 (0.003) <0.001

Intercept 25(OH)D

Lowest 0.016 (0.004) <0.001 0.015 (0.004) <0.001 0.011 (0.004) 0.007

Middle (ref) 0.0 0.0 0.0

Highest -0.004 (0.005) 0.49 -0.003 (0.005) 0.51 -0.003 (0.005) 0.56

Slope 25(OH)D X Time n/a

Lowest X Time 0.002 (0.005) 0.64 0.002 (0.004) 0.67

Middle X Time (ref) 0.0 0.0

Highest -0.003 (0.006) 0.96 -0.0005 (0.005) 0.93

___________________________________________________________________________________________________________________________________________

Supplementary Table 3 (continued) ___________________________________________________________________________________________________________________

Outcome Effects Model 1 *P* Model 2 *P* Model 3 *P*

β (SE) β (SE) β (SE)

_____________________________________________________________________________________________________________________________

PoA Time 0.025 (0.003) <0.001 0.023 (0.005) <0.001 0.023 (0.004) <0.001

Intercept 25(OH)D

Lowest 0.029 (0.007) <0.001 0.030 (0.005) <0.001 0.022 (0.007) 0.002

Middle (ref) 0.0 0.0 0.0

Highest 0.0002 (0.009) 0.98 -3.531E-5 (0.009) 1.00 -0.001 (0.009) 0.93

Slope 25(OH)D X Time n/a

Lowest X Time 0.009 (0.008) 0.26 0.009 (0.008) 0.24

Middle X Time (ref) 0.0 0.0

Highest -0.005 (0.01) 0.58 -0.005 (0.01) 0.58

__________________________________________________________________________________________________________________

RTV Time 0.004 (0.004) 0.36 0.004 (0.005) 0.49 0.004 (0.005) 0.40

Intercept 25(OH)D

Lowest 0.026 (0.008) 0.001 0.028 (0.009) 0.001 0.023 (0.009) 0.01

Middle (ref) 0.0 0.0 0.0

Highest -0.003 (0.01) 0.79 -0.007 (0.011) 0.53 -0.006 (0.011) 0.59

Slope 25(OH)D X Time n/a

Lowest X Time -0.006 (0.009) 0.52 -0.004 (0.009) 0.67

Middle X Time (ref) 0.0 0.0

Highest 0.011 (0.011) 0.36 0.010 (0.011) 0.37

__________________________________________________________________________________________________________________

CoA Time 0.064 (0.043) 0.14 0.086 (0.057) 0.13 0.104 (0.054) 0.06

Intercept 25(OH)D

Lowest 0.315 (0.086) <0.001 0.321 (0.092) 0.001 0.292 (0.090) 0.001

Middle (ref) 0.0 0.0 0.0

Highest -0.124 (0.108) 0.25 -0.084 (0.116) 0.32 -0.054 (0.112) 0.63

Slope 25(OH)D X Time n/a

Lowest X Time -0.017 (0.101) 0.87 -0.011 (0.096) 0.91

Middle X Time (ref) 0.0 0.0

Highest -0.112 (0.124) 0.37 -0.121 (0.117) 0.30

________________________________________________________________________________________________________________________________________

SRT, Simple Reaction Time; CRT, Choice Reaction Time; DVT, Digit Vigilance Reaction Time; PoA, Power of Attention; RTV, Reaction Time Variability; CoA, Continuity of Attention

^a^Estimated β values (SE) of fixed effects using transformed longitudinal data for all outcomes. Random effects terms included both intercept and slopes of attention scores over time. Time in the study was coded as baseline (0), 1.5-y follow-up (1) and 3-y follow-up (2). ^b^Serum 25(OH)D was categorized in season-specific quartiles. Middle quartiles (SQ2 and SQ3) were combined and served as the reference group.

Model 1 includes serum 25(OH)D and liner trend of time.

In Model 2 a linear trend of time by serum 25(OH)D interaction is added.

Model 3 is further adjusted for education, sex, smoking status, current alcohol intake, renal impairment, and number of chronic diseases (0-1 diseases (0), 2 diseases (1), 3 and more diseases (2)).

**Supplementary Figures**

**
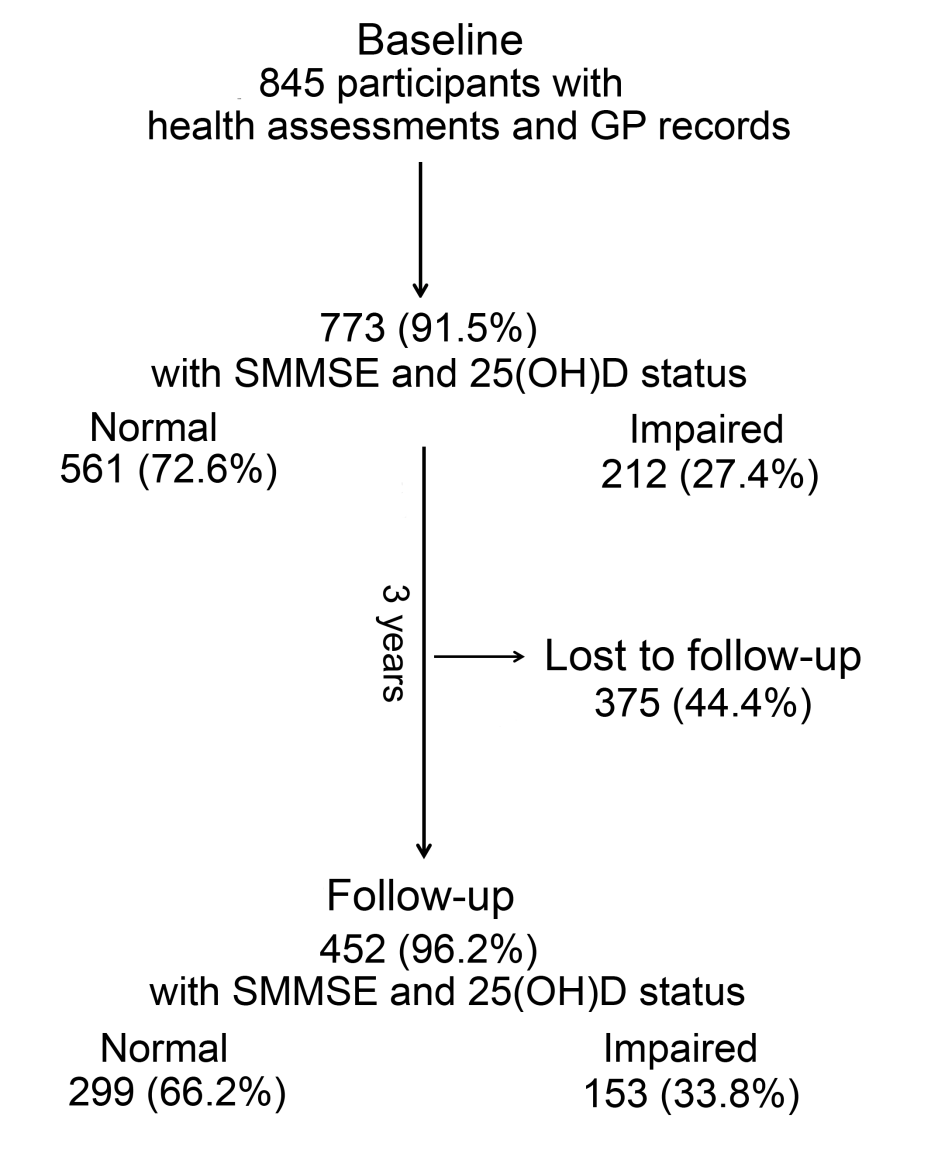
**

**Supplementary Figure 1** Newcastle 85+ Study participants by SMMSE scores and 25(OH)D status availability over the study period.
